# A Deep Learning Method for Near-Real-Time Cloud and Cloud Shadow Segmentation from Gaofen-1 Images

**DOI:** 10.1155/2020/8811630

**Published:** 2020-10-29

**Authors:** Mehdi Khoshboresh-Masouleh, Reza Shah-Hosseini

**Affiliations:** School of Surveying and Geospatial Engineering, College of Engineering, University of Tehran, Tehran, Iran

## Abstract

In this study, an essential application of remote sensing using deep learning functionality is presented. Gaofen-1 satellite mission, developed by the China National Space Administration (CNSA) for the civilian high-definition Earth observation satellite program, provides near-real-time observations for geographical mapping, environment surveying, and climate change monitoring. Cloud and cloud shadow segmentation are a crucial element to enable automatic near-real-time processing of Gaofen-1 images, and therefore, their performances must be accurately validated. In this paper, a robust multiscale segmentation method based on deep learning is proposed to improve the efficiency and effectiveness of cloud and cloud shadow segmentation from Gaofen-1 images. The proposed method first implements feature map based on the spectral-spatial features from residual convolutional layers and the cloud/cloud shadow footprints extraction based on a novel loss function to generate the final footprints. The experimental results using Gaofen-1 images demonstrate the more reasonable accuracy and efficient computational cost achievement of the proposed method compared to the cloud and cloud shadow segmentation performance of two existing state-of-the-art methods.

## 1. Introduction

Cloud and cloud shadow are among the causes of disruption in processing passive sensors' images in remote sensing [1]. The presence of cloud and cloud shadow in remote sensing images disrupts the processes that involve segmentation, classification, matching, and the production of 3D models [2–4]. The accurate detection of cloud and cloud shadow is a significant step in the multispectral image preprocessing [5]. The most important studies on the cloud detection, such as the Global Cloud Monitoring Project [6], have used an Advanced Very High-Resolution Radiometer (AVHRR), AVHRR processing program for ice, snow, and cloud monitoring [7], and the International Satellite Cloud Climatology Project [8] has used the thermal channel data with low spatial resolution.

Monitoring Earth using the high-spatial resolution remote sensing images has been of great interest during the recent years. Most remote sensing satellites with high spatial resolution imaging have limited spectral channels (e.g., red, green, blue, and near-infrared) due to device considerations [9]. The remote sensing images with limited spectral channels, such as Gaofen-1 images, often lack complete radiometric calibration parameters due to the absence of the thermal and the water vapor absorption channels [10]. The process of identifying clouds accurately and separating them from some features, i.e., coastlines or buildings, is highly complicated [11]. In this context, providing a solution to detect and eliminate clouds and cloud shadows from images with high spatial resolution in different scenes is of great importance. The process of eliminating the cloud and cloud shadow from images depends on the accuracy of the cloud and cloud shadow detection [12]. To improve the accuracy of cloud and cloud shadow detection in high-spatial resolution images, several studies have been carried out using the statistical methods of pattern detection [13], common methods of machine learning such as support vector machines [14], and deep learning methods [15–18]. The results of recent studies using deep learning methods on visible and near-infrared channels images of Zi-Yuan 3 satellites with a spatial resolution of 5.8 meters, Gaofen-1 with a spatial resolution of 16/8 meters, and Gaofen-2 with a resolution of 4 meters indicate an improved cloud detection accuracy with a mean accuracy of 92%, while revealing the margin details of clouds and cloud shadows due to various complications at this level of image resolution is still a significant challenge [19, 20].

In this study, a new method based on the deep convolutional neural network was proposed for automatic near-real-time cloud and cloud shadow segmentation from Gaofen-1 satellite images. The proposed method is based on the theory of recurrent and deep convolutional networks in a multiscale structure. The most important innovations and contributions to the development of problem solving are as follows:

- A deep convolutional neural network with a multiscale structure was presented for the better segmentation of the marginal details of clouds and cloud shadows from other complications.
- The design of residual convolutional blocks based on the depth dropout method in the multiscale structure aimed at reducing calculation costs and improving the accuracy of segmentation results was another innovation of this study.
- In this study, a weighted cross-entropy function was used for solving the imbalance of target pixels.
- The comparison of the proposed method with an advanced statistical method and an advanced deep learning method which aimed at the automatic clouds and cloud shadows detection had the best results using Gaofen-1 satellite images.

The remainder of the manuscript is organized as follows: In the next section, the related works are briefly reviewed.  Section 3 presents the details of the proposed method and data used. The results are analyzed and discussed in Section 4. Finally, the conclusions drawn from this study are elaborated in Section 5.

## 2. Related Works

Signal and image processing systems can play a key role in real-world applications, such as vehicle collision avoidance, microdrilling monitoring, and many engineering projects. In this regard, Castaño et al. [21] introduced a new self-tuning method for increased obstacle detection reliability based on Internet of Things Light Detection and Ranging (LiDAR) sensor models. In this method, a density-based spatial clustering of application with a noise (DBSCAN) algorithm was applied [22] for 3D point cloud segmentation, which can segment the 3D point cloud for each available obstacle at the scene. Beruvides et al. [23] proposed a study about the correlation between the holes quality and the force signals in the microdrilling process in a sintered tungsten-copper alloy.

In recent decades, researchers have conducted extensive studies on the cloud and cloud shadow detection using different data of remote sensing as a single scene or multitemporal scenes, including Moderate Resolution Imaging Spectroradiometer (MODIS) images [24], Landsat series images [25], and Sentinel-2 images [26]. The methods used in previous studies can be classified into two classes as follows. The first class includes the statistical methods of pattern recognition and the process of cloud detection and, sometimes, cloud shadow detection based on brightness temperature through the thermal channels of remote sensing images by determining the threshold value. The MODIS cloud mask [27], FMask algorithm optimization (Presented by U.S. Geological Survey) for Sentinel-2 images [28], MAJA method (presented by the French Space Agency) for Landsat and Sentinel-2 multitemporal scenes [29, 30], and the Sen2Cor processor (Presented by the European Space Agency) for Sentinel-2 images [31] are some examples of statistical methods for detecting clouds and cloud shadows being proposed in the recent decades. The methods in the first class have no good function in the cloud and cloud shadow detection from the images with high spatial resolution because of the lack of thermal channels and a problem in threshold value due to high spatial resolution [32]. The second class involves machine learning methods. Machine learning methods based on training data often perform the process of the cloud and cloud shadow detection with desirable accuracy. Shallow artificial neural network methods for Landsat single scene images [33], support vector machines for WorldView-2 images [34], and object-based machine learning methods for Gaofen-1 images [35] are among the machine learning methods. Machine learning methods have played a more effective role in cloud detection than the images with high spatial resolution, while the conventional methods have no acceptable accuracy. Using deep learning methods (in this study, deep convolutional neural networks are considered), which are among the complete subsets of machine learning methods, has been highly regarded in remote sensing image processing [36, 37]. One of the fundamental needs of deep learning methods is the need for big data [38, 39]. In the field of remote sensing, deep learning methods have a good performance in different fields of remote sensing due to the presence of big data. Another challenge in deep learning is an appropriate infrastructure for data processing. Developing cloud computing infrastructures for deep learning, such as the Google Colab Service, is one of the best solutions of using deep learning in image processing studies. The results of analyzing the research background in the field of the cloud and cloud shadow detection of the images with high-spatial resolution include the following:

- Previous methods were often developed from the initial architecture of deep learning such as U-Net [40] or SegNet [41]. The initial architectures of deep learning are currently less considered in image processing due to the lack of optimal structure and multiple uses of the same layers of convolution without any justification.
- Based on the results of previous studies, as the spatial resolution is higher, the ability of algorithms in near-real-time cloud and cloud shadow detection is significantly reduced [42]. However, the variety of processed scenes has highly affected the performance of deep learning algorithms. In other words, in studies where clouds and cloud shadows have been considered in different scenes such as water zones, agricultural lands, or snow-covered regions, the overall accuracy of detection has decreased.
- The studied areas in the previous studies often included the areas with homogeneous levels [43]. Thus, regarding the cloud and cloud shadow detection in different areas in the images of the satellites such as Gaofen-1 is of great importance.
- Based on the results of previous studies, as the spatial resolution is higher and the scenes are more complex, the overall accuracy of the cloud and cloud shadow detection decreases significantly.

## 3. Proposed Method

In this study, the proposed method is a new architecture in deep learning. The most significant features of the proposed method include the following:

- Using convolutional filters with different dimensions in an encoding/decoding structure for better separation of the marginal details of clouds and cloud shadows from other terrestrial features is one of the innovations of this study
- Designing the residual convolutional blocks based on the deep dropout method, unlike conventional residual blocks that operate in the identical way
- Developing a cross-entropy loss function for increasing the uniformity and equilibrium among pixels and improving the accuracy of the cloud and cloud shadow detection is one of the initiatives of this algorithm

**Figure 1:**
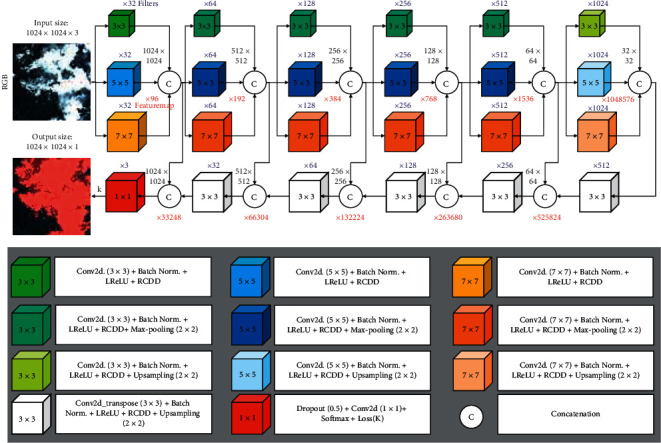
The proposed network for cloud and cloud shadow segmentation. “Conv2d (*k *×* k*)” stands for the convolutional kernel with the size of *k *×* k*; “Batch Norm.” denotes batch normalization; “LReLU” denotes the leaky rectified linear units; and “RCDD” denotes the residual convolutional layer based on depth dropout.

Figure 1 illustrates the architectural structure of the proposed method. The proposed algorithm is an architecture with an end-to-end learning process. The end-to-end learning process refers to learning all the extracted features in the model training process and testing it without any postprocessing method. The proposed architecture is a deep learning architecture with a network depth of 6 (network length). In addition, a unique innovation was used in this network instead of convolutional layers with constant filter dimensions consecutively such as U-Net or SegNet networks. This innovation includes the use of the filters with different dimensions for the training process. Based on the results of this study, using the filters with different dimensions in the convolutional neural networks has the following advantages:

- This network generates new features and automatically integrates them along with the network, which is an appropriate method for reinforcing the data (Figure 1 displays the number of features in each layer in red and the number of filters in blue).
- The access to global-local features is provided simultaneously. For example, if the objective is extracting cloud shadows, there may be some structures in the shadow part, and if a filter with fixed dimensions is used, the structures will cause a disturbance in the extraction process. However, bigger filters can used to eliminate their effect in this area.

Our criterion for determining the network depth and the number of layers with various filter dimensions is the maximum Random Access Memory (RAM) available in the Google Colab environment. In other words, based on infrastructural limitations, the network has developed the most in terms of length (network depth) and width (number of filters with different dimensions). In this study, implementing the proposed method was performed using the Python programming language and deep learning open-source programming library called Keras [44], being developed in the Python language in the Google Colab cloud computing environment. One of the features of using the Keras library is running deep learning models on the Tensorflow processing unit. The Tensorflow processing unit is the strongest processor for deep learning studies which can be used in the Google Colab cloud computing environment.

### 3.1. Residual Convolutional Layer Based on Depth Dropout (RCDD)

**Figure 2:**
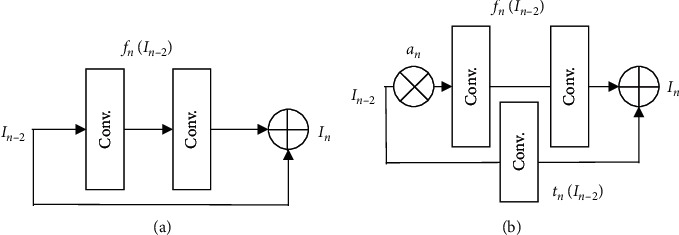
Schemes are illustrated for different residual convolutional layers based on (a) the general structure and (b) depth dropout (RCDD).

Using the residual blocks in deep learning architectures, despite the improvement of classification accuracy, significantly increases the cost of calculations. The increased cost of calculations significantly affects the integration of the residual blocks with the convolutional layers. Assuming that the convolutional block includes two convolutional filters, the input value passes through two filters and, then, is added to the initial value (Figure 2(a)). As the theory suggests, the processed value is added to the initial value merely for preventing the reduction of the features created by the convolutional filters. This process makes it difficult to employ these blocks in deep encoding-decoding architectures. In order to promote the use of residual blocks in deep encoding-decoding architectures, a new method called convolutional residual blocks based on deep dropout method was used in this study (Figure 2(b)). Deep dropout method was used for the first time for 56-layer ResNet network with the aim of classifying objects on the CIFAR10 and ImageNet sets (two image sets known in computer sciences) [45].

The results indicated an increase in processing speed up to 17.5% in comparison to the normal 56-layer ResNet structure. In general, a typical residual block is calculated using equation (1). However, the residual block is determined based on the deep dropout method using equation (2).

(1) $$\begin{array}{c} I_{n}=f_{n}\left(I_{n-2}\right)+I_{n-2}, \end{array}$$

(2) $$\begin{array}{c} I_{n}=a_{n}f_{n}\left(I_{n-2}\right)+t_{n}\left(I_{n-2}\right), \end{array}$$

where *I*_*n*_ represents the residual block output; *f*_*n*_ represents the transfer function of convolution. In addition, *I*_*n*−2_, an, and *t*_*n*_ represent the residual block input, generalization scale following the Bernoulli distribution, and training function (in the initial form of the random descending gradient).

In this study, a major change was made in the structure of the deep dropout method to develop this method for the convolutional filter. Such a change includes the use of the comparative moment estimation training method instead of the random reduction gradient training method. Since the random reduction gradient method is a method with high computational cost and the training process is not optimized properly, a new and optimal method of estimating the comparative moment, the performance of which was studied in many studies, was used. Hyperparameters in the comparative moment estimation method are considered as 0.01, 0.9, 0.999, and 10^(−8)^ for the learning rate, beta-1, beta-2, and epsilon, respectively. In addition, the *he_norm* method is used for initializing the network. The *he_norm* method is one of the most appropriate weighting methods in deep learning [46, 47].

### 3.2. Weighted Cross Entropy

The role of an appropriate loss function in deep convolutional neural networks is of great importance [48–50]. Because of the large amount of training data in deep learning, many loss functions have poor performance because of the incorrect (even low) data. Most deep learning methods used in previous studies have used the least squares error (L2) for calculating the network error. One of the main disadvantages of using the least squares error in deep convolutional neural networks is the weak performance for dealing with incorrect data. The data used in remote sensing are not often without any error (such as noise in image), and the segmentation issues have often an imbalance in target pixels, thus designing an appropriate loss function is inevitable. In the field of image segmentation, increasing the uniformity of the loss function and creating a balance between the target and nontarget pixels improves the architectural performance in the updating process and final output by using deep learning methods (such as cloud and cloud shadow detection). The updating process is often based on gradients. For this purpose, if the loss function has more uniformity, the derivation process is facilitated for achieving an optimal training (optimal convergence of error values).

The first cross-entropy method was introduced for probability estimation of rare events. In this regard, Haber et al. [51] introduced a new multiobjective optimization based on the cross-entropy method with only four parameters. In this study, the weighted pattern of the cross-entropy function was used for developing the cross-entropy error function. In the weighted cross-entropy function, a weight close to one is considered for all target pixels. This is obtained by dividing the number of nontarget pixels by the total number of pixels, which is called the equilibrium parameter. In this study, it is necessary to use the equilibrium parameter since the number of nontarget pixels is higher in some samples. The weighted cross-entropy function for multiclass classification is defined as follows:

(3) $$\begin{array}{c} H{\left(p,\;\hat{p}\right)}_{k}=-\left(\beta \;\log\left(\hat{p}\right)+\left(1-p\right)\text{log}\left(1-\hat{p}\right)\right), \end{array}$$

where *H* represents the output value in the known class; *k* represents the class type (cloud class, cloud shadow class, and nontarget class). *p*, $\hat{p}$, and *β* represent the probability condition for target output, probability condition for non-target output, and equilibrium parameter, respectively. The loss function designed at the end of the proposed architecture is used for classifying the predicted classes.

### 3.3. Dataset

**Figure 3:**
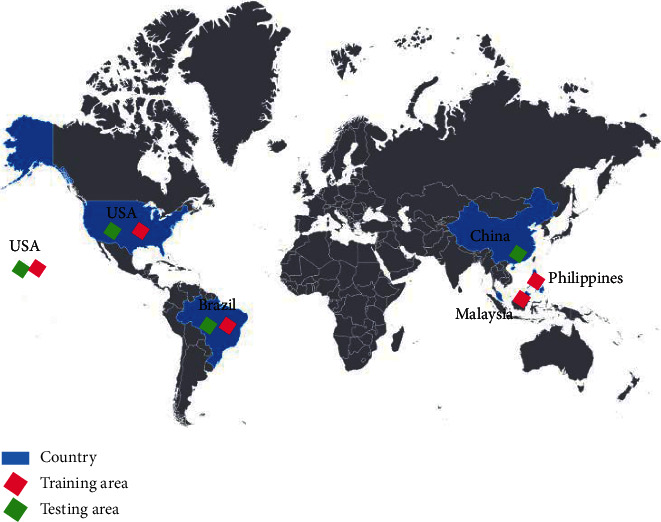
Distribution of the training and testing study areas (base-map credit: Ref [53]).

In this study, 100 images taken by the Gaofen-1 satellite with Red-Green-Blue composite images at dimensions of 1024 × 1024 pixels and 2-A products were used for evaluating the proposed method. The level of 2-A products in the Gaofen-1 satellite involves the products with partial radiometric correction and systematic geometric correction. The used images are a subset of [13]. The ground truth for clouds and cloud shadows was prepared by a specialized human agent using the proposed method in [52]. In order to evaluate the distribution of training and experimental samples in the research data set, Figure 3 is presented.

In order to increase the reliability of results in terms of the generalizability of the proposed method, the used set of images has the following challenges:

- The used images were taken during 2013–2016. The variety of time for taking the images leads to various clouds in different seasons of the year.
- The used images were provided from different cities in China, the Philippines, Malaysia, the USA, and Brazil.
- The used images in different scenes are agricultural lands, water zines, coastal regions, and semiurban areas.

## 4. Results and Discussion

The condition for gaining the highest accuracy among the previous methods is an appropriate criterion for selecting reference methods for comparative study using the proposed method. Two state-of-the-art methods, including Fast Multifeature Combined (Fast-MFC) [13], and Multiscale Convolutional Feature Fusion (MSCF) [54], were used for comparisons. Because of their known efficiency in cloud and cloud shadow segmentation from Gaofen-1 satellite images, these methods were selected. The Fast-MFC and MSCF were tested in this study from the beginning, using the same testing set that was applied for the testing of the proposed method.

### 4.1. Fast-MFC

Fast-MFC is a statistical method of pattern recognition for cloud and cloud shadow detection from Gaofen-1 satellite images based on the Mean Absolute Error (MAE) and the Mean Relative Error (MRE). The first step of this method is implementing a threshold for segmentation based on spectral features and segmentation refinement based on a guided filter (a bilateral filter for improving the edge) in order to generate the cloud initial range. Then, the geometric features are combined with the texture features for improving the results of cloud detection and the final production of cloud maps. Eventually, cloud shadow maps are extracted by matching the clouds and cloud shadows.

### 4.2. MSCF

MSCF is a new deep learning method based on cross-entropy loss and mean-squared error loss for cloud and cloud shadow detection from different remote sensing images, especially the images with high spatial resolution, such as Gaofen-1 satellite images. This method is about 2% more accurate than other results compared to previous methods of deep learning such as DeepLab and DCN, which have been used for cloud and cloud shadow detection. In addition, this algorithm has been tested in different scenes compared to previous studies. This method proposed a deep convolutional encoding-decoding architecture.

### 4.3. Accuracy Metrics

In the field of the cloud and cloud shadow detection of the images with high-spatial resolution, the accurate evaluation of the location of the cloud and cloud shadow pixels is of great importance. Thus, the criteria which are defined based on the accuracy of pixels have many applications. In this study, two criteria were considered for accuracy evaluation, including the *F*1 score and the Intersection over Union (IoU). Based on the recent studies, using the *F*1 score and the IoU provides an appropriate evaluation of the validation of the results in terms of the cloud and cloud shadow detection [15]. The *F*1 score indicates an average criterion between the accuracy and sensitivity obtained from the results. In other words, a kind of average is regarded based on the accuracy of the predicted data and the ratio of the predicted data to the total data. The *F*1 score is calculated based on the error matrix values as follows:

(4) $$\begin{array}{c} F1\text{ score}=\frac{2\times \text{TP}}{\left(2\times \text{TP}\right)+\text{FP}+\text{FN}}. \end{array}$$

The IoU describes the similarity or difference in the set of desired samples. This criterion calculates the ratio of the two sets' similarity to the number of the two sets. The IoU is currently one of the most widely used and reliable criteria in evaluating the image segmentation results. The IoU is calculated based on error matrix values as follows:

(5) $$\begin{array}{c} \text{IoU}=\frac{\text{TP}}{\text{TP}+\text{FP}+\text{FN}}, \end{array}$$

where TP represents the number of cloud pixels (or cloud shadow) in the cloud class (or cloud shadow); FP represents the number of cloud pixels (or cloud shadow), in the noncloud class (or noncloud shadow); and FN indicates the number of noncloud pixels (or noncloud shadow) in the cloud class (or cloud shadow).

### 4.4. Experimental Results

**Figure 4:**
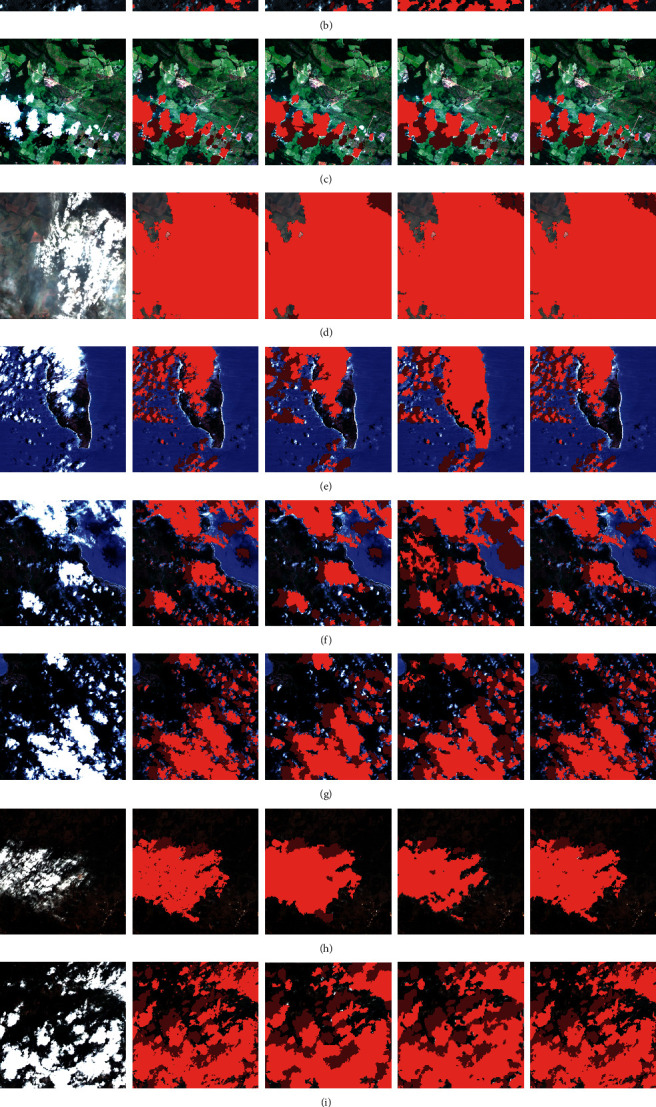
Visual comparison with the Fast-MFC, the MSCF, and the proposed method across ten images (light red: cloud; and dark red: cloud shadow).

**Figure 5:**
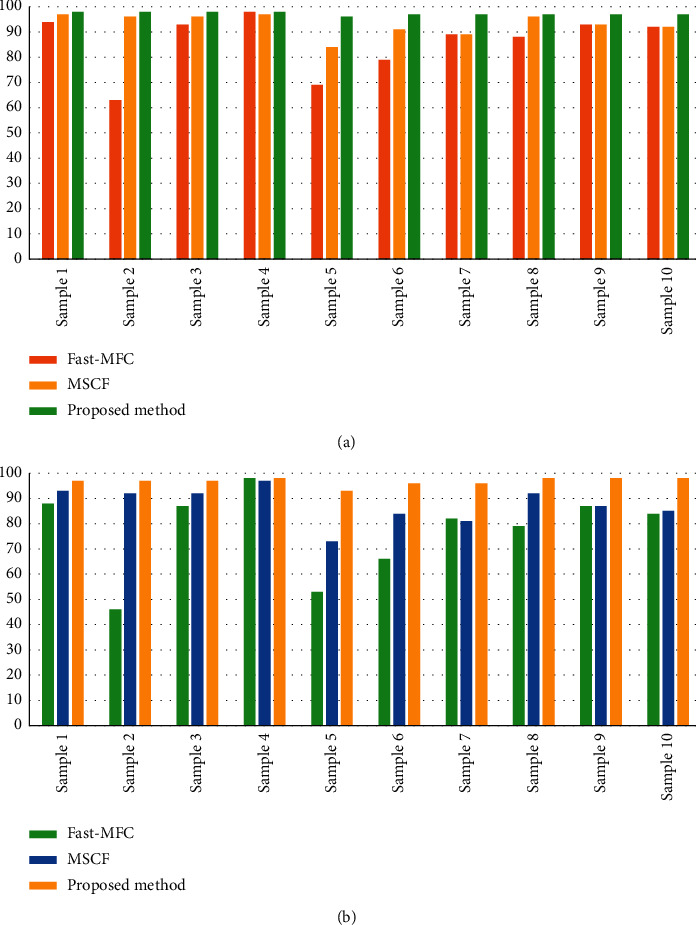
Accuracy assessment of the cloud segmentation for different sample images with the Fast-MFC, the MSCF, and the proposed method. (a) Mean *F*1 score. (b) Mean IoU.

**Figure 6:**
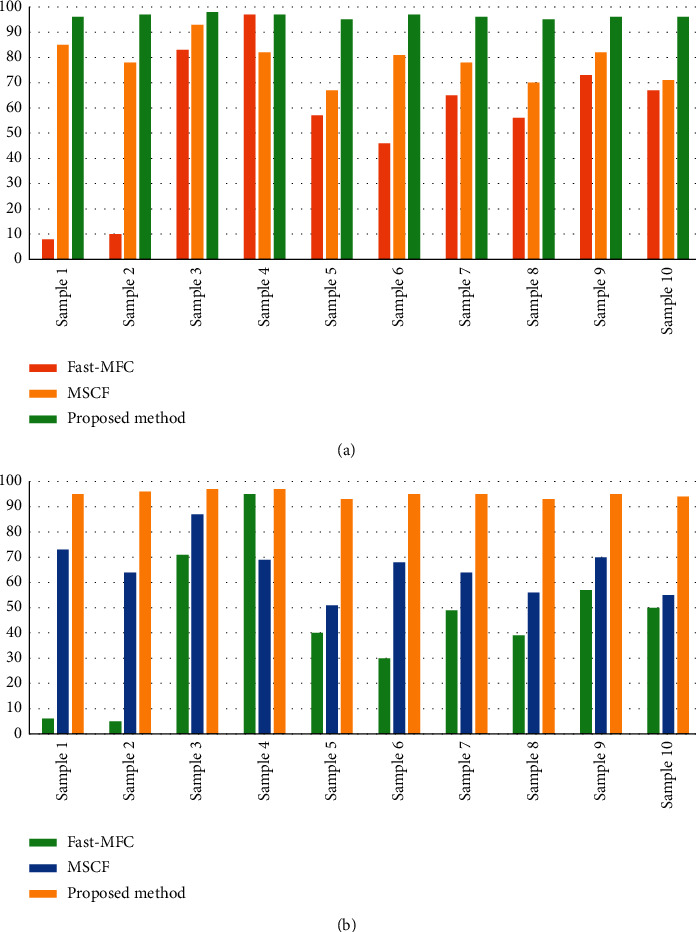
Accuracy assessment of the cloud shadow segmentation for different sample images with the Fast-MFC, the MSCF, and the proposed method. (a) Mean *F*1 score. (b) Mean IoU.

Figure 4 shows the cloud and cloud shadow segmentation results of the different methods. Figures 5 and 6 display the numerical results of the test performed using the proposed architecture, compared to the reference methods. In this study, ten different samples were selected for a challenging test. The results obtained from the test by the proposed method, the Fast-MFC method as a statistical algorithm, and the MSCF method as a deep learning algorithm are as follows.

#### 4.4.1. Evaluating the Accuracy of the Methods in Cloud Segmentation

  The proposed method was improved about 15% and 25% in the F1 score criteria and the IoU compared to the Fast-MFC method
  The proposed method was improved about 8% and 15% in the F1 score and the IoU compared to the MSCF method
  The MSCF method had a better performance of about 10% and 17% in the *F*1 score and the IoU compared to the Fast-MFC method

All three methods used in cloud segmentation have an acceptable performance, while the Fast-MFC and MSCF methods have no appropriate performance while facing small clouds (such as the second, sixth, and seventh samples). Since the spatial resolution of the Gaofen-1 satellite images equals 16/8 meters, small cloud detection is of importance and the proposed method has had a good performance.

#### 4.4.2. Evaluating the Accuracy of Methods in Cloud Shadow Segmentation

  The proposed method was improved about 47% and 56% in the *F*1 score and the IoU compared to the MFC method
  The proposed method was improved about 22% and 31% in the *F*1 score and the IoU compared to the MSCF method
  The MSCF method had a better performance of about 30% and 29% in the *F*1 score and the IoU compared to the MFC method

The Fast-MFC method had no good performance compared to the proposed method and MSCF methods in cloud shadow detection and, sometimes, detected the first, second, fifth, ninth, and tenth samples, as well as the cloud and cloud shadows, wrongly. The MSCF method had a weaker performance than the proposed method in partial cloud shadow detection (e.g., Figures 4(e)–4(h)).

#### 4.4.3. Evaluating the Accuracy of Methods in Challenging the Presence of Dense Fog in Scene

The fourth example (Figure 4(d)) in this test has the challenge of the presence of dense fog in scene. Since the purpose of creating an algorithm for the cloud and cloud shadow detection is using these results in the recovery phase of the effects covered by clouds and cloud shadows, dense fog was considered in the cloud class in this test.

The Fast-MFC method and the proposed method have better results compared to the MSCF method. The results indicated that the Fast-MFC method, as a statistical method, had a desirable performance in distinguishing between the dense and sparse fog and cloud shadows.

The objective of using deep learning methods is achieving the same function as the expert human factor with the least error. The proposed method had a good performance in cloud detection in the challenge of the presence of dense fog in scene. In addition, cloud shadows were identified accurately in this challenge.

#### 4.4.4. Processing Cost

Calculating the cost of processing is raised between the proposed method and the MSCF method as a deep learning method. Processing cost refers to the time spent on training deep learning architecture and its criterion is based on the number of repetitions of the time spent for each repetition. The processing costs for the proposed method and the MSCF method for 200 repetitions of training equal 2000 and 2570 minutes, respectively. The proposed method has a less computational cost of about 570 minutes compared to the MSCF method.

#### 4.4.5. Comparative Studies Using SVM and Fuzzy k-Means

**Figure 7:**
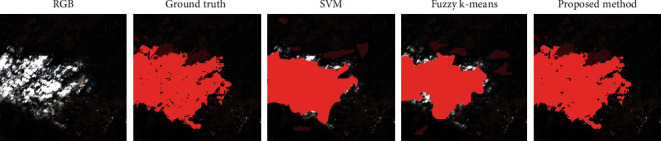
Accuracy assessment of the cloud shadow segmentation with the SVM, the Fuzzy k-means, and the proposed method.

For the benchmark, in addition to the Fast-MFC and MSCF, two effective methods, including Support Vector Machine (SVM) [55, 56] and Fuzzy k-means [57], were used for comparisons. The qualitative comparison of the proposed method prediction with SVM and Fuzzy k-means can be seen in Figure 7. The qualitative results show the ability of the proposed method to segment smaller cloud/cloud shadow regions in scene while producing a perfect result of the overall scene.

## 5. Conclusions and Further Research

This study presents a novel multiscale deep learning method for near-real-time cloud and cloud shadow segmentation using Gaofen-1 images. The proposed model performs well and is comparable in accuracy to existing the cloud and cloud shadow segmentation methods that were developed for Gaofen-1 images. In addition, the proposed model was applied to extract the cloud and cloud shadow for ten scenes of crucial challenges in remote sensing. The advantage of the proposed method is that it takes into account the spectral-spatial relationship of the multiscale data and eliminates the need to consider additional parameters for its task.

In the future study, the proposed method can be extended for real-time applications. Our future research will address real-time cloud and cloud shadow segmentation in the different sensors.

## Data Availability

The public open-source dataset used to support this study is available at http://sendimage.whu.edu.cn/en/mfc/.
